# Memory consolidation from seconds to weeks: a three-stage neural network model with autonomous reinstatement dynamics

**DOI:** 10.3389/fncom.2014.00064

**Published:** 2014-07-01

**Authors:** Florian Fiebig, Anders Lansner

**Affiliations:** ^1^Department of Computational Biology, Royal Institute of Technology (KTH)Stockholm, Sweden; ^2^Institute for Adaptive and Neural Computation, School of Informatics, Edinburgh UniversityEdinburgh, Scotland; ^3^Department of Numerical Analysis and Computer Science, Stockholm UniversityStockholm, Sweden

**Keywords:** memory consolidation, working memory, complementary learning systems, synaptic depression, neural adaptation, retrograde amnesia, anterograde amnesia, retrograde facilitation, computational model

## Abstract

Declarative long-term memories are not created in an instant. Gradual stabilization and temporally shifting dependence of acquired declarative memories in different brain regions—called systems consolidation—can be tracked in time by lesion experiments. The observation of temporally graded retrograde amnesia (RA) following hippocampal lesions points to a gradual transfer of memory from hippocampus to neocortical long-term memory. Spontaneous reactivations of hippocampal memories, as observed in place cell reactivations during slow-wave-sleep, are supposed to drive neocortical reinstatements and facilitate this process. We propose a functional neural network implementation of these ideas and furthermore suggest an extended three-state framework that includes the prefrontal cortex (PFC). It bridges the temporal chasm between working memory percepts on the scale of seconds and consolidated long-term memory on the scale of weeks or months. We show that our three-stage model can autonomously produce the necessary stochastic reactivation dynamics for successful episodic memory consolidation. The resulting learning system is shown to exhibit classical memory effects seen in experimental studies, such as retrograde and anterograde amnesia (AA) after simulated hippocampal lesioning; furthermore the model reproduces peculiar biological findings on memory modulation, such as retrograde facilitation of memory after suppressed acquisition of new long-term memories—similar to the effects of benzodiazepines on memory.

## Introduction

Memories for facts and events are not acquired in their definite form. Rather, some post-learning processes are known to take place that gradually stabilize new memories. It is generally accepted that neocortex provides a slow learning substrate for distributed long-term memories. Fast working memory, capable of immediate one-shot learning, has been localized around the PFC (Jacobsen, 1936; Fuster, 2008). The discourse on declarative memory consolidation has, however, been largely centered around the hippocampus and various substructures of the wider medial temporal lobe (MTL), which store memories on an intermediate timescale, and thus are perfectly situated to moderate the consolidation process.

Any eventual declarative long-term memory existed at its earliest stage in PFC as working memory, next in the MTL, and finally in the hippocampally-independent neocortical long-term memory, so multiple brain areas are thought to support declarative memory throughout its lifetime. As memory is transitionally stored in memory systems of very different capacity and plasticity, a holistic model of declarative memory must find a way to interlink the involved networks functionally, using the available biological data about phenomenology as well as anatomical structures and neurophysiology.

In this paper, we will focus on mechanistic systems level modeling of this remarkable feature of human memory, namely the enormous temporal chasm (seconds to decades) bridged by the memory consolidation process and the neural mechanisms behind it.

After a brief Introduction of the Complementary Learning Systems (CLS) framework and biological evidence for consolidation through reactivations/replay, we lay down four challenges, which we see as important to address in modeling memory consolidation.

The Model and Method section introduces our three-stage concept, then the formal network model, followed by the full memory consolidation model with its different components and simulation cycle. We establish our performance metric and present our method for simulating hippocampal lesions.

In the Results section, we highlight some key simulation results including autonomous memory consolidation, lesion-induced amnesia effects and two memory modulation experiments, which follow a range of different memory phenomena typical to the mammalian, declarative memory system.

After attempting to validate our computational memory consolidation model by contrasting it against biological evidence in this way, we discuss the broader implications this has for the CLS framework and future computational memory consolidation models, as well as contradictory biological evidence and possible augmentations of the model.

### Origins of the CLS framework

The study of memory systems consolidation has resulted in several computational and neural network models of increasing refinement (McNaughton and Morris, 1987; Alvarez and Squire, 1994; Wilson and McNaughton, 1994; McClelland et al., 1995; Shen and McNaughton, 1996; McClelland, 1998; Hasselmo and McClelland, 1999; Wittenberg et al., 2002; Norman and O'Reilly, 2003; Walker and Russo, 2004; Roxin and Fusi, 2013), which have largely confirmed the idea that a composition of multiple interacting learning systems is both useful and necessary for replicating many aspects of human memory including recognition memory data.

The hippocampus was established to play a major role in the process of memory consolidation most notably by the case of Patient HM (Milner, 1972) and various animal lesion studies by Zola-Morgan et al. (Squire and Zola-Morgan, 1985, 1991; Zola-Morgan and Squire, 1990; Squire, 1992). Patients with lesions not only exhibit severe anterograde amnesia (AA) but also temporally graded retrograde amnesia (RA), primarily affecting recent—not yet consolidated—memories (Zola-Morgan and Squire, 1985). Non-declarative types of memory, such as priming, motor, or perceptual learning are not affected by hippocampal lesioning and are thought to be reliant on other brain regions and mechanisms.

Functionally, structures of the MTL memory system, in particular the hippocampus, are believed to form an anatomical index. Distributed neocortical activations of an event are thereby bound together into a coherent memory trace or encoded in a more suitable form than the neocortical activation itself, achieving strong pattern separation and recall performance. High plasticity in the hippocampus facilitates fast learning while granting the neocortex the time necessary to integrate new memories into the preexisting structure of older long-term memories. With progressing systems consolidation, memories become hippocampally independent over time.

It has been suggested that working memory performance may be aided by hippocampus/MTL, especially for relational processing (Olson et al., 2006; Graham et al., 2010), but more recent studies reveal that working memory performance remains unaffected by hippocampal and even wider MTL lesions if the capacity requirements of the task do not exceed a narrowly defined working memory capacity (Jeneson et al., 2010; Jeneson and Squire, 2012). This evidence lends itself to two conclusions: First, working memory itself is independent of the hippocampus. Second, the hippocampus may still aid working memory by extending the available capacity.

Increasingly precise hypotheses (Eichenbaum et al., 2011) about functionally distinct roles of different structures surrounding the hippocampal area (e.g., perirhinal cortex and parahippocampal region) in recollection vs. familiarity and in encoding direct or indirect relationships between items and contexts, warrants the use of the wider term MTL rather than treating intermediate memory function as a mere hippocampal issue. However, the scope of analysis for this paper rests on associative recall and discussion of larger brain area interactions, so here we refrain from a detailed breakdown of MTL subareas.

Based on the theoretical consideration of incremental learning in artificial neural networks (McClelland et al., 1995; McClelland, 1998), it was concluded that the existence of at least two CLS appears to be necessary. Such a two-stage CLS serves an adaptive function and allows for processes of selective learning, memory strength modulation, and gradual acquisition into stable long-term memory without sacrificing one-shot learning capability.

Most memory models concerned with hippocampal-neocortical interaction (e.g., Alvarez and Squire, 1994; McClelland et al., 1995; Murre, 1996; Wittenberg et al., 2002) account for the different time-course of memory formation in hippocampus and neocortex by assuming fast synaptic plasticity in hippocampus and much slower, gradual modifications in neocortex. Attractor states are quickly learned in the hippocampal network and then later used to spread components of the association in the neocortex. In this view, hippocampus effectively acts as a teacher to neocortex and has also been described as a training-trial-multiplier (Norman et al., 2005).

Sleep and its various phases have been proposed to modulate network dynamics and plasticity, thus promoting this supposed two-phase memory consolidation process (Wilson and McNaughton, 1994; Qin et al., 1997; Buzsáki, 1998), whereby interference between new learning (awake) and consolidation (asleep) is avoided. Especially with respect to sequential memories, recurring reactivations have also been called replay.

### Reactivation/replay

Spontaneous reactivations (or replay) have repeatedly been observed in the hippocampus, but also in other brain areas, such as PFC (Euston et al., 2007; Peyrache et al., 2009). Large ensembles (Louie and Wilson, 2001; Lee and Wilson, 2002) of place cells in the rat hippocampus were found to reactivate during REM-sleep and particularly slow-wave sleep (SWS) in a consistent sequential order similar to prior wake state activations. Especially SWS reactivations were shown to co-occur with brief (30–120 ms), irregular sharp-waves/ripples (SWR) at 100–250 Hz in the local field potential (Buzsáki et al., 1983; Buzsáki G Horváth et al., 1992; Buzsáki, 1986). During a SWR event, a small fraction of neurons in the CA3-CA1 subicular complex/entorhinal cortex discharge synchronously in powerful population bursts (Sullivan et al., 2011). The resulting neural events might reach far away to associated cortical areas to induce LTP. The number of reactivation events have been repeatedly linked to memory performance in many tasks such as spatial learning (Dupret et al., 2010), odor-reward association learning, and retrieval from remote memory (Eschenko et al., 2008). The amnesic effects of targeted replay interruption via electrical stimulation (Girardeau et al., 2009; Ego-Stengel and Wilson, 2010) suggest that this link is causal, not merely correlational.

### Four challenges to modeling

In the following, we lay out four major challenges which we see as critical for the advancement of a more complete model of memory consolidation and aim to address with our model.

#### Autonomous replay

Despite the fact that reinstatement is a critical component of the supposed consolidation process, surprisingly few neural network models (Norman et al., 2005) concerned with memory consolidation consider how an artificial neural network might be adapted such that continuous replay activity becomes an emergent system property, and could be harnessed for autonomous long-term memory consolidation dynamics in hippocampal-neocortical interaction. The basic problem can be described like this:

Attractor neural networks are commonly used to store memories in computational models of cortical memory (Lansner, 2009). Such an approach is justified on the grounds of observation of attractors in hippocampus and neocortex. For example, the rich collateral connectivity in the hippocampal CA3 region can be modeled as an associative feedback matrix (Marr, 1970, 1971; McNaughton and Morris, 1987; Treves and Rolls, 1994). This usually results in fixed-point attractor dynamics, and the major issue with modeling replay under these conditions is that activity is inherently stable once it has converged. Consequently, most computational models of the consolidation process impose a scheme of repeated random noise bursts (Murre, 1996; Wittenberg et al., 2002; Walker and Russo, 2004; Roxin and Fusi, 2013), predetermined activation patterns (Alvarez and Squire, 1994), or externally regulated subcortical disinhibition (Bibbig, 1996), designed to take the system out of its current attractor state and thus cue the reactivation of another previously learned attractor. Often, even papers specifically concerned with modeling “*spontaneous reactivatio*n” do not implement an intrinsic neural mechanism for spontaneous reinstatement, but use noise.

We believe that computational memory models need to include a functional and biologically plausible intrinsic mechanism of replay that can facilitate autonomous replay and thus drive consolidation. Consequently the model presented in this paper uses an attractor network capable of autonomous replay, describes some of its characteristics and uses these to functionally drive a consolidation mechanism.

#### Inclusion of working memory

As of today, the CLS framework has no account of working memory and its many implementations—successful as they may be in other respects—have thus notoriously neglected it in the modeling effort. This is unfortunate, as hippocampal (or MTL, as the model may have it) memory trace formation is consequently assumed to be automatic, near instantaneous (i.e., one-shot learning), and largely synonymous with working memory when it comes to acquisition (Norman, 2010). Even simple word list learning demonstrates, however, that not every fleeting percept automatically acquires a lasting episodic memory trace in HIP/MTL supporting recall. Serial position effects in these kinds of memory tests (primacy and recency), first described by Hermann Ebbinghaus at the end of the Nineteenth Century, reveal a time-dependent consolidation process at work in the formation of a lasting memory trace susceptible to attention, relevance, and conscious reflection. Only then can the consolidated hippocampal trace itself later drive long-term systems consolidation into neocortex. Each network effectively acts as a teacher to the next and in this sense, we aim to test the viability of a consolidation-chain, comparable to more theoretical multi-stage network models recently proposed by Roxin and Fusi (2013). We believe that the inclusion of working memory into CLS, in whatever fashion, is a critical step toward addressing the issue. Toward this goal, we implement a very fast learning network of exceedingly limited capacity (supporting recall of about five to seven recent items/attractors), mimicking pre-frontal working memory functionality.

#### Temporal scope of systems consolidation

Biological data on the time course of systems consolidation is abundant in RA and AA gradients following hippocampal lesioning (Winocur, 1990; Kim and Fanselow, 1992) and studies on humans with impaired MTL (Zola-Morgan et al., 1986; Jeneson et al., 2010). Many neural network models of memory exist, replicating numerous aspects of human memory, yet the full temporal scope of memory consolidation from working memory to long-term memory has not been addressed adequately.

We believe this is in large part because it is hard to model mechanistically. The temporal scales on which working memory, intermediate memory, and long-term memory operate are separated by many orders of magnitude in time. On-line learning rules for artificial neural networks used in memory modeling need to reflect this in their time constants. A further complication is simulation runtime: Even without significant scaling (toward biologically reasonable network size), simulations of systems consolidation spanning weeks or months almost immediately result in prohibitively long simulation runtimes, especially if neural dynamics are simulated at the resolution of a few milliseconds.

We believe the temporal scope of real memory needs to be addressed in computational modeling attempts. With this objective in mind, we implement plasticity time constants ranging from minutes to days, which may not cover the needed span entirely, but allows a comparison with actual learning/amnesia curves in rodents (see Figure 9) and is meant as a serious step toward such a memory system.

#### Catastrophic forgetting

Catastrophic Forgetting (CF) is a common problem in attractor memory networks. Without special attention to the learning rule, the tendency of many kinds of neural networks is to eventually forget previous information abruptly upon learning new information. As such, CF is a radical manifestation of the so called stability-plasticity dilemma. While the principled division of labor proposed by the CLS model improves the trade-off between stability and plasticity drastically, as networks can specialize in either high stability or high plasticity, it still cannot fundamentally solve the problem by itself. A dedicated stable, long-term network with large capacity will delay the onset of CF, but at its core, the network learning rule must allow the network to forget as dynamically as it learns or CF will eventually become a problem.

We believe that a functional memory system should be able to learn and forget indefinitely and that addressing CF is critical in improving the biological plausibility of artificial neural networks for human memory models. Toward this goal, our model implements a memory process that can keep learning/forgetting indefinitely and effectively addresses the issue of CF from a theoretical and functional vantage point.

## Model and methods

### Three-stage model

**Figure 1:**
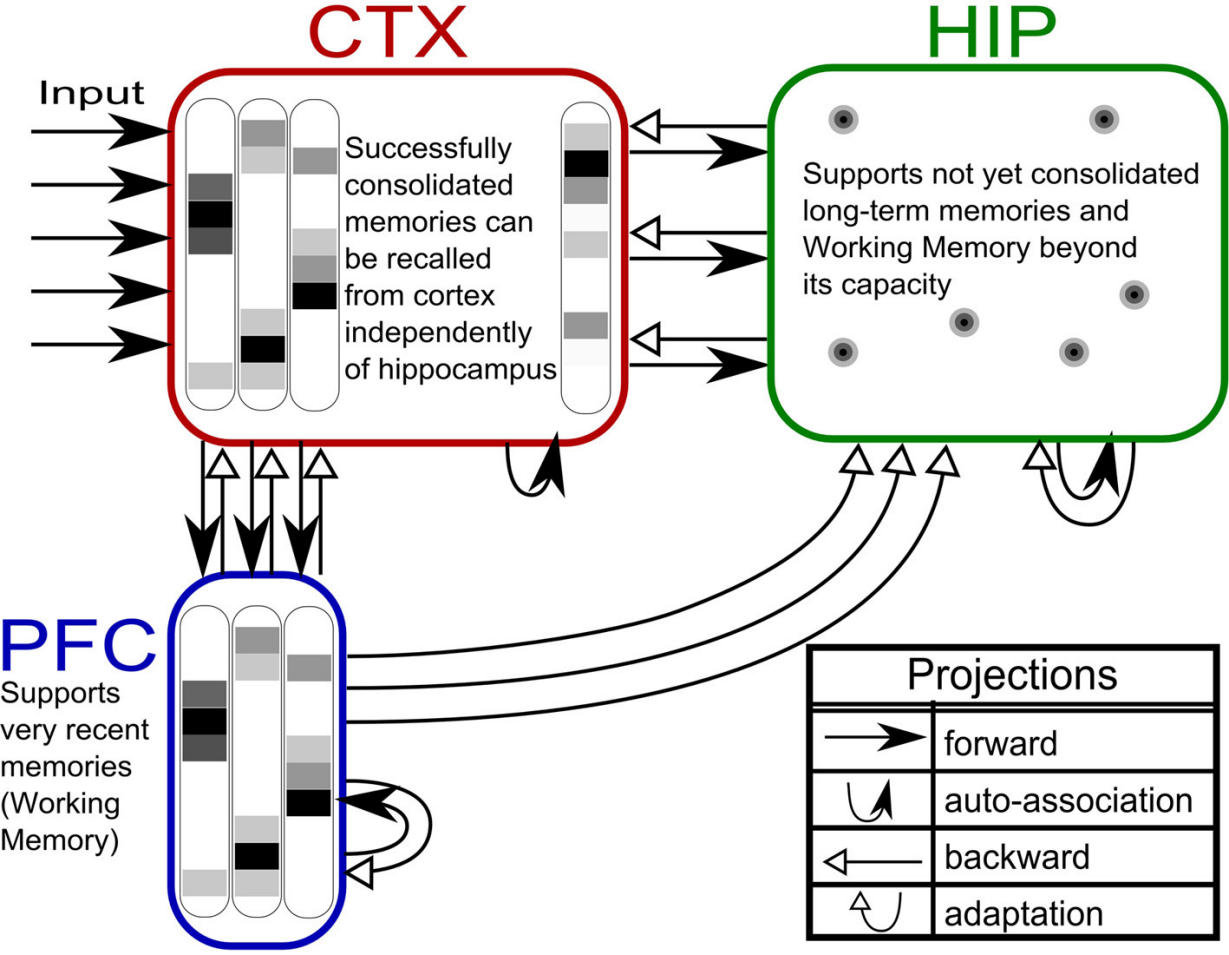
**The three-stage memory model: from prefrontal short-term memory to long-term neocortical memory**. Activity in cortical areas (PFC, CTX) is organized into hypercolumns, while HIP activity is sparser, pattern-separated, and lacks columnar organization.

Based on our own previous work and inspired by the CLS framework, (McClelland et al., 1995; Norman et al., 2005; Norman, 2010) we built a three-stage memory system (Figure 1), also incorporating hippocampally independent and more short-lived working memory. The formal model will be described in the next section, detailed network and simulation parameters can be found in Tables 1, 2. Time constants are estimations rather than being based on neurobiological data. In that sense, they constitute model predictions.

**Table 1:** **Network parameters**.

**Parameter**	**Symbol [unit]**	**PFC**	**HIP**	**CTX**	**PFC-to-HIP**	**HIP-to-CTX**
Network size	N	50	250	500		
Number of hypercolumns	H	5	–	50		
Activity level (sparsity)	A	10%	5%	10%		
Learning time constant	τ_L_ [ms]	8	400	18,500	20	20
-corresponds to (scaled):		3 [min]	3 [h]	6 [days]	9 [min]	9 [min]
Association gain	g_L_	1	1	1	1	1
Adaptation time constant	τ_A_ [ms]	160	400	–	–	–
Adaptation gain	g_A_	–1.2	–0.8	–	–	–
Recall detection threshold^*^	Θ	0.093	0.252	0.383	–	–
**SIMULATION PHASES**						
**Learning phase name**	**Perception**		**Reflection**		**Sleep**	
Length	3 [steps]		52 [steps]		165 [steps]	
-corresponds to (scaled):	13 [min]		3.8 [h]		12 [h]	
**GENERAL PARAMETERS**						
Membrane time constant	τ_C_ [ms]					1
Intrinsic noise rate	λ_0_					0.025
Hypercolumn size	M_i_ [units]					10

* *Near zero recall rates of unstudied patterns 352–360 (0 Days old in Figure 7) demonstrate that the classification thresholds on the recall distance are not too loose, generating barely any false positives*.

**Table 2:** **Projection parameters during different simulation phases**.

		**Perception**	**Reflection**	**Sleep**	**Recall**
PFC	g_L_	1	1	0	1
	τ_L_ [ms]	5	∞	5	∞
	g_A_	−1.2	−1.2	0	0
	τ_A_ [ms]	120	120	∞	∞
	g_PFC2HIP_	0	1	0	0
	g_PFC2CTX_	0	1	0	0
HIP	g_L_	1	0	1	1
	τ_L_ [ms]	400	400	∞	∞
	g_A_	−0.8	0	−0.8	0
	τ_A_ [ms]	400	∞	400	∞
	g_HIP2CTX_	0	0	1	0
CTX	g_L_	1	0	0	1
	τ_L_ [ms]	18.500	18.500	18.500	∞
	g_CTX2PFC_	1	0	0	1
	g_CTX2HIP_	1	0	0	1

*Note that infinite time constants denote no learning of this projection during that particular phase, e.g., no learning occurs during recall*.

The first population, modeling the PFC, has the smallest size (50 units) but features the fastest learning with a time-constant τ_*L*_ = 3 min. This design is supposed to mimic short-term memory and comprise the substrate for working memory as well: A rapid memory system, capable of learning from single examples, but forgetting equally fast, resulting in highly limited capacity. It should be noted that the hypothesized short-term memory mechanism is synaptic rather than of a more standard persistent activity type. It is based on fast expressing and volatile Hebbian synaptic plasticity and modulated intrinsic excitability (Sandberg et al., 2003; Mongillo et al., 2008). Current biological data on fast forms of synaptic plasticity as well as intrinsic excitability modulation suggest that such a mechanism of short-term memory is indeed a possibility (Fransén et al., 2006; Lee et al., 2009; Lansner et al., 2013). This suggests that the widely different temporal characteristics of cortical memory systems are mainly due to plasticity with a corresponding spectrum of time constants. This network uses a kind of columnar coding, which is described in the next section.

The second population (250 units), modeling the intermediate-term hippocampal memory system (which might anatomically involve close-by areas of the MTL such as the perirhinal cortex and parahippocampal area), is five times larger and much slower learning with time-constant τ_*L*_ = 3 h. This network is modeled without hypercolumns and in this case, a k-winner-take-all (kWTA) mechanism is used to produce a sparse and distributed representation, this is further described in the Pattern Representation section.

The last population (CTX) models a large (500 units) and slow learning (τ_*L*_ = 6 days) neocortical long-term memory, with columnar structure. It is obviously hard to teach a memory system this slow learning anything without either massive repetition, or internal reinstatement dynamics. Note, that without the use of additional metaplasicity in synaptic learning (Fusi et al., 2005), time constants probably need to span this wide range to even approach biologically plausible timescales between working memory and stable long-term memory.

### Formal model

We use an auto-associative Bayesian Confidence Propagation Neural Network (BCPNN) (Sandberg et al., 2002, 2003) with adapting non-spiking units modeling cortical minicolumns representing a local sub-population of some 100 neurons (Buxhoeveden and Casanova, 2002). These are further bundled into soft-winner-take all (soft-WTA) modules referred to as hypercolumns (Kanter, 1988; Favorov and Diamond, 1990). A normalizing lateral feedback inhibition within the hypercolumn is assumed to be mediated by inhibitory basket cells. Previous studies of this type of modular network have demonstrated their excellent functional capabilities as associative memories (Johansson and Lansner, 2007a,b) including the ability to replicate primacy, recency, and serial recall effects in human immediate free recall (Lansner et al., 2013). We have further shown that when we replace the abstract non-spiking units in such a network with more biophysically detailed spiking model neurons, we can successfully reproduce several experimental key phenomena in memory recall, like nested oscillatory dynamics and spontaneous reactivation (Lundqvist et al., 2006, 2010, 2011). All units are connected with associative weights (stored in weight matrix *w*), using incremental Hebbian learning with a time constant τ_*L*_ (Sandberg et al., 2002) which can be varied to accommodate different levels of plasticity. Cellular adaptation and depressing synapses were modeled by use of an additional projection between neurons with a negative gain and its own learning time constant τ_*A*_, which was given a value of 160 ms. This projection abstractly models both the decay rate of slow after-hyperpolarization in a previous biophysically detailed pyramidal cell model (Fransén and Lansner, 1995; Sandberg and Lansner, 2002) and synaptic depression on the same time scale (Markram et al., 1997; Lundqvist et al., 2006). Cellular adaptation and synaptic depression are prominent features of biological cortical pyramidal cells (Adelman et al., 2012) and synapses connecting pyramidal cells in cortex (Lanting et al., 2013).

The network is simulated in time steps of 10 ms. Each unit *i* belongs to a hypercolumn of size *M*, and *H*(*i*) defines the set of units in the same hypercolumn. The support *h* of each unit is computed via the update Equation (1), where *g_L_* denotes the gain of the auto-associative projection and *g_A_* denotes the gain of the adaptation projection. Values for these and other model parameters are found in Table 1. The output, ${\hat{\pi }}_{j}(t)$ of these units, a measure for neural activity, is then computed in Equation (2), which also achieves the aforementioned hypercolumnar normalization. In Equations (3)–(4), the current activity is used to update rate estimates for units Λ_*i*_ and connections Λ_*ij*_. Through temporal filtering with a learning time constant, these represent heuristically estimated probabilities which are consistent with prior information. These running average rate estimates are then used to compute bias β, as well as synaptic weights *w* in Equations (5)–(6). While this paper cannot motivate the entire derivation of the BCPNN learning rule, it should not go unmentioned that these equations were originally derived from a naive Bayesian classifier (so the weight is a joint activity rate estimate divided by the unit rate estimates). A minimal noise background activity λ_0_, impacts how strong/weak the correlation measures between units (as encoded by the weights) can become. It essentially guarantees an upper and lower bound on the weight, avoids underflow (as we use the logarithmic weight during the update) and weight stability in the absence of input. The membrane time-constant τ_*c*_ is set to 1. The adaptation bias γ and adaptation weights *v* are activity dependent as well and the exact same Hebbian-Bayesian learning rule applied to the original associative projection is used for the adaptation projection (Equations 7–10)—with the important distinction that adaptation acts on a different timescale, so the rate estimates μ_*i*_ and μ_*ij*_ are computed on the timescale of τ_*A*_.

(1) $$\begin{array}{l} \tau_{C}\frac{dh_{j}(t)}{dt}=g_{L}\left[\beta_{j}(t)+\sum_{k}\log\left(\sum_{i\epsilon H(k)}^{M_{k}}w_{ij}(t){\hat{\pi }}_{i}(t)\right)\right]\\ \;+g_{A}\left[\gamma_{j}(t)+\sum_{k}\log\left(\sum_{i\epsilon H(k)}^{M_{k}}v_{ij}(t){\hat{\pi }}_{i}(t)\right)\right]-h_{j}(t) \end{array}$$

(2) $${\hat{\pi }}_{j}(t)\;=\frac{e^{h_{j}}}{\sum_{j\epsilon H(j)}e^{h_{j}}}$$

(3) $$\tau_{L}\frac{d\Lambda_{i}(t)}{dt}\;={\hat{\pi }}_{i}(t)-\Lambda_{i}(t)$$

(4) $$\tau_{L}\frac{d\Lambda_{ij}(t)}{dt}\;={\hat{\pi }}_{i}(t){\hat{\pi }}_{j}(t)-\Lambda_{ij}(t)$$

(5) $$\beta_{i}(t)\;=\;log\left(\Lambda_{\text{j}}(t)\right)$$

(6) $$w_{ij}(t)\;=\frac{\left(\text{1}-\lambda_{0}^{2}\right)\Lambda_{ij}(t)+\lambda_{0}^{2}}{\left[\left(\text{1}-\lambda_{0}\right)\Lambda_{i}(t)+\lambda_{0}\right]\left[\left(\text{1}-\lambda_{0}\right)\Lambda_{j}(t)+\lambda_{0}\right]}$$

(7) $$\tau_{A}\frac{d\mu_{i}(t)}{dt}\;={\hat{\pi }}_{i}(t)-\mu_{i}(t)$$

(8) $$\tau_{A}\frac{d\mu_{ij}(t)}{dt}\;={\hat{\pi }}_{i}(t){\hat{\pi }}_{j}(t)-\mu_{ij}(t)$$

(9) $$\gamma_{i}(t)\;=log\left(\mu_{\text{j}}(t)\right)$$

(10) $$v_{ij}(t)\;=\frac{\left(\text{1}-\lambda_{0}^{2}\right)\mu_{ij}(t)+\lambda_{0}^{2}}{\left[\left(\text{1}-\lambda_{0}\right)\mu_{i}(t)+\lambda_{0}\right]\left[\left(\text{1​}-\text{​}\lambda_{0}\right)\mu_{j}(t)+\lambda_{0}\right]}$$

As BCPNNs learn probability estimates of internal and external events, their activity flow, ${\hat{\pi }}_{j}(t)$, can be interpreted as inference. BCPNNs exhibit unequal coding strength for learned patterns, depending on the overlap with other learned patterns and most importantly, their age. The dynamical, gradual forgetting of the oldest patterns allows BCPNNs to learn new patterns indefinitely and escape the problem of CF that haunts other kinds of neural networks and often necessitates some process of interleaved unlearning to keep these networks viable for memory modeling of this kind (Walker and Stickgold, 2004).

**Figure 2:**
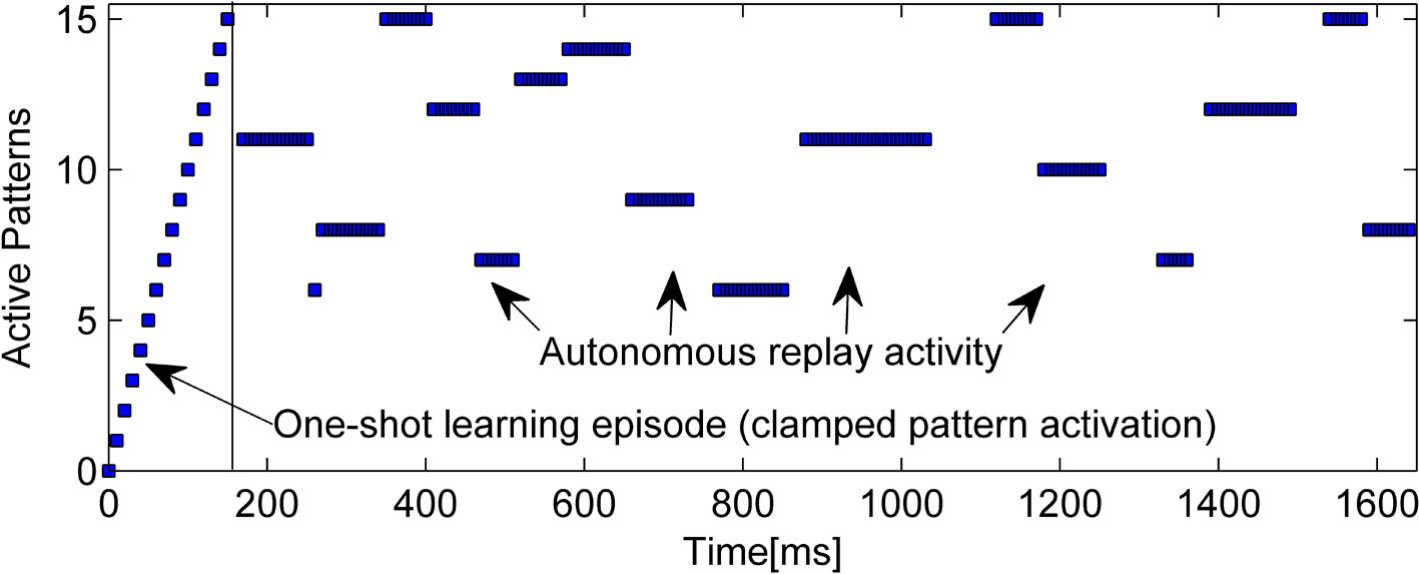
**Example of learning (15 random patterns, sequentially trained over 150 ms) and subsequent autonomous replay activity in a small BCPNN (50 Units in 5 Hypercolumns)**. Note that some early patterns are never replayed due to forgetting, while other, stronger patterns reactivate multiple times. Longer reactivations are often a sign of less correlated patterns.

An example of the intrinsic replay activity generated by this kind of network can be seen in Figure 2.

### Pattern representations

Having multiple involved brain regions entails multiple memory traces that may coexist but serve the same episodic memory. The randomly drawn neocortical input patterns have one active unit per ten-unit hypercolumn and consequently randomly varying degrees of overlap, a major cause of the model's stochastic behavior.

The three memory systems are inter-connected by feed-forward and feed-back connections. There are several possible ways of setting up these connections. Neurobiologically, the internal representations of the connected structure are expected to differ. While sensory activations in earlier cortical processing stages are expected to represent specific stimulus properties, HIP and PFC activations likely represent abstracted, sparsified, and decorrelated versions of such internal representation.

**Figure 3:**
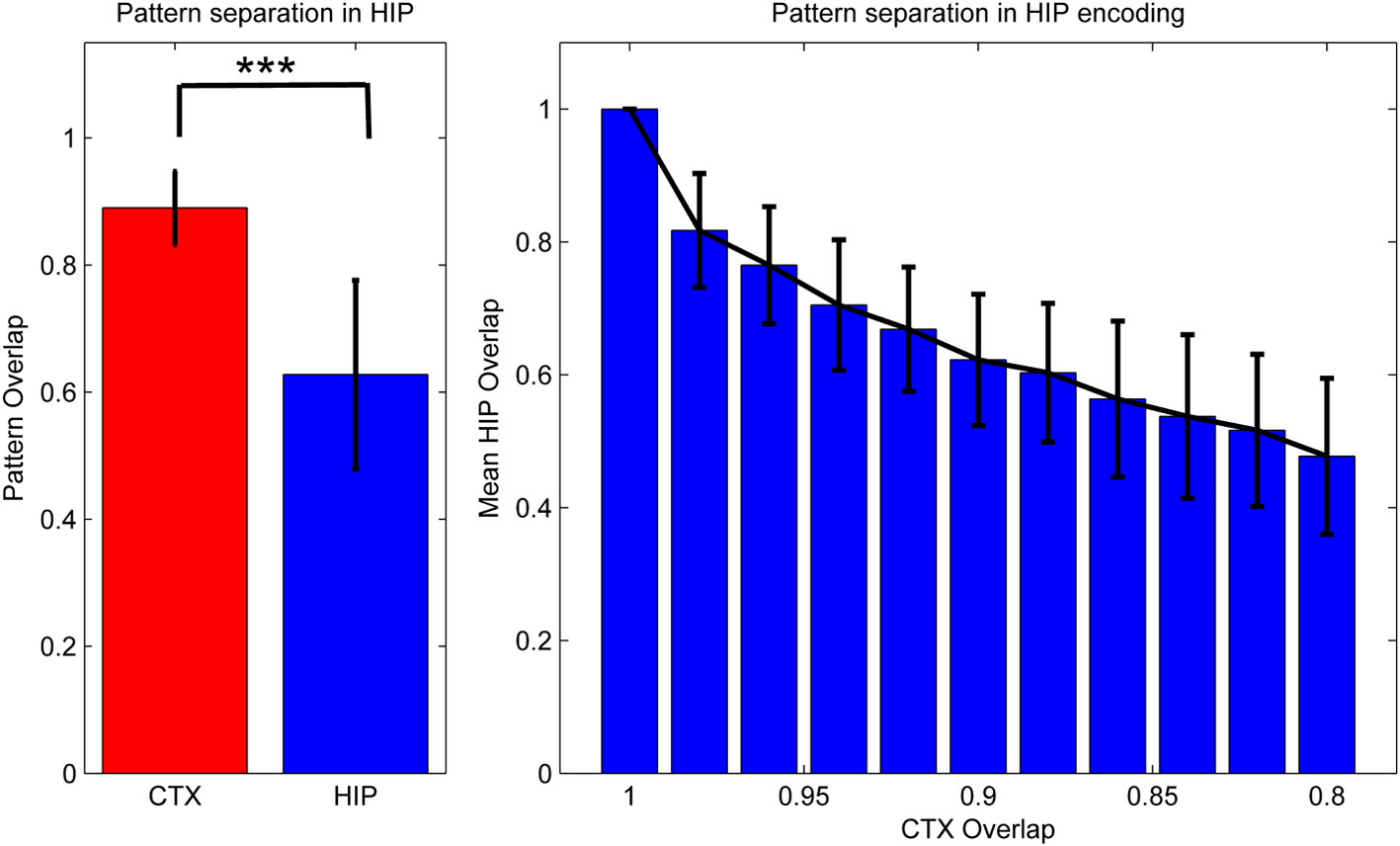
**Activity in HIP changes with the cortical input, as measured by normalized activity overlap**. The hippocampus implements a pattern separation mechanism which yields highly significant changes in activity when cortical input changes only slightly. **Left**: randomly varying the activity of 1–10 of the 50 cortical hypercolumns yields highly significant changes in hippocampal encoding. Note that to highlight the variability of coding, error bars denote one STD, not SEM (^***^We test against H_0_: median difference between the pairs is zero. As normal distributions of overlaps are not guaranteed in this case, we use the non-parametric pairwise Wilcoxon signed-rank test, yielding *p* < 10^−165^, when using 1000 pairs.) **Right**: effective pattern separation can also be seen from the fact that hippocampal patterns diverge much faster than cortical input, e.g., changing the activity of just one cortical hypercolumn yields a 2% CTX pattern change (as measured by 98% overlap), but nearly 17% in HIP. When we change 10 hypercolumns (80% cortical overlap) then about half of the originally active HIP units are no longer a part of the encoded pattern. Note again that error bars denote STD, rather than SEM.

For reasons of simplicity, we assumed PFC patterns to be a subset of the CTX patterns generated through a 1-to-1 connection between units that leaves out some CTX units, as PFC has fewer units (A HIP → PFC connection can in principle be implemented to derive some of the PFC activation from HIP activity as well, but was left out here in favor of a more transparent generation of training pattern activity). For the HIP representation however, the forward connection from CTX is implemented as a sparsification-process (kWTA) that reduces the level of activity by half (to 5%) and achieves strong pattern separation. In the pattern generator, this is implemented by connecting the CTX activation to HIP through a connection matrix with random, constant weights and selecting the 5% most active units (i.e. *k* = 13) as the derived hippocampal encoding of that pattern. The practical implication of such an implementation is that if two CTX inputs are becoming less similar, the HIP representations of these input patterns will quickly become much more dissimilar, assigning distinct representations to each input pattern (Figure 3), while the respective CTX (and PFC) representations will on average be similar (as measured by normalized pattern overlap) to the same degree as the input. This is justified qualitatively by experimental observations of sparse activation and strong pattern separation in Dentate Gyrus and CA3 (Leutgeb et al., 2007; Bakker et al., 2008), while the quantitative choice of doubling sparsity is an arbitrary choice that seems to work well.

As previous memory models emphasizing the importance of hippocampal pattern separation have noted, this coding scheme lends HIP quite different operating characteristics than CTX, namely a positive Y-Intercept in the ROC curve (Norman, 2010). While others have stressed that these findings can be used to explain differences in modes of recognition (recall vs. familiarity), we have found additional benefits for our model: increased pattern separation makes HIP not just better at discriminating between studied items and related lures (while sacrificing some capability to compute global match), but also improves replay performance in our model, because it reduces ambiguity/overlap and thus allows for strong reinstatements, which are—after all—key to successful systems consolidation.

The real process of feed-forward input abstraction, compression, or decorrelation presumably occurs through bi-directional connections between the different network modules. Regarding the back-projections, some consolidation models simply use a static 1-to-1 connection (Wittenberg et al., 2002) or random subsets of such (Murre, 1996) to connect these structures. Instead, we used plastic connections in the back-projections with a fast learning time constant τ_*L*_ = 9 min. This enables our model to learn associations between arbitrary representations, allowing for different coding in separate brain areas/stages.

### Simulation phases

**Figure 4:**
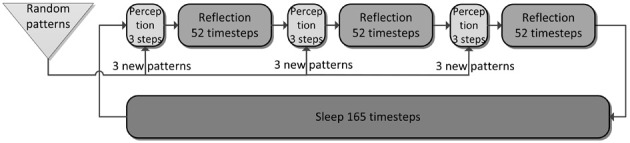
**The simulation cycle with its three alternating phases, named perception, reflection, and sleep**. Online learning occurs only during perception. All other learning is a function of memory consolidation during reflection and sleep. The gating of various projections at the transition between simulation phases is summarized also in Table 2.

**Figure 5:**
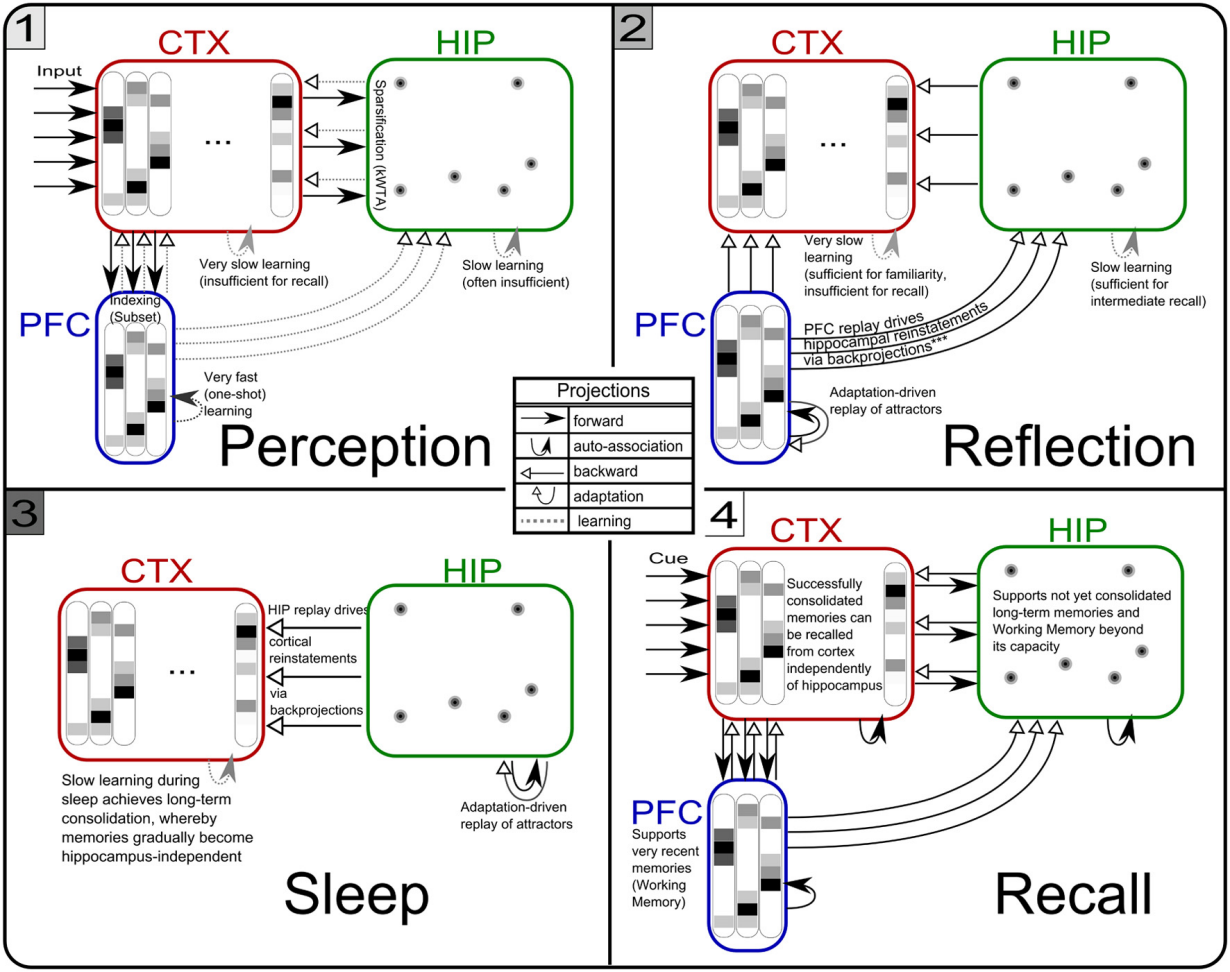
**The three simulation phases 1–3 and their active components, as well as the configuration during cued recall after many days of consolidation**. During perception, feed-forward projections from neocortical input generate separate PFC and HIP traces, which are associated to the CTX trace via Hebbian-learning in the back-projections. This online learning episode is very brief and effectively too short to establish lasting HIP and CTX memories. During the reflection phase, replay in PFC (similar to active rehearsal) generated by the interplay of its auto-association and adaptation projections, drives HIP reinstatements, thus facilitating learning in its auto-associative projections. During sleep, HIP replay then drives CTX reinstatements which facilitate long-term learning. During cued recall, the external neocortical activation generates corresponding cues in PFC and HIP through feed-forward connections. All three networks are then individually or simultaneously allowed to relax/converge to attractors, potentially yielding successful recall of a corresponding training pattern. ^***^It should be noted, that the strongest influence of the PFC on the hippocampus in primates is indirect through parahippocampal cortices. The direct projection PFC-to-HIP is neuroanatomically non-existent (Otani, 2004). We consider this modeling issue in the discussion.

Our simulation evolves in three phases (Figures 4, 5) plus one phase for recall testing afterwards, during which plasticity is turned-off. Apart from initial brief online learning (using clamped CTX activity) and modulation of network-dynamics (gains and time constants) imposed at the transition of phases, no external intervention in the dynamic activity was undertaken. Most importantly, the learning networks stayed plastic during the cycles of convergence and gradual depression of projected patterns, as opposed to models that selectively wait for complete convergence of attractors before executing any learning rules (Murre, 1996; Wittenberg et al., 2002).

We ultimately want to show the consolidation performance of the overall memory system. For that reason, we made the original online learning episodes, called perception, very brief. Each new training pattern is shown for only one simulation time step, forcing one-shot learning in PFC, as the other networks learn too slowly for recall after this short exposure. Consolidation is then achieved through spontaneous reactivation of learned patterns, which in turn causes the corresponding patterns to be projected in the next network and thus potentially learned or strengthened.

As we undertake a full simulation cycle of one day and one night in just 330 time steps (Figure 4) of 10 ms each, the model plasticity is scaled against reality by a factor of roughly 26.000. The chief motivation for this is to enable a study of systems of this kind at all: Without temporal scaling of this sort, simulation of weeks or months becomes infeasible due to runtime considerations. At this scaling, the chosen time constants of 3 min (Short-term memory), 3 h (Intermediate-term memory) and 6 days (Long-term memory), are mapped onto 8, 400, 18,500 ms respectively, thus preserving the ratio of timescales mapped out by the choice of time constants. Note, that this is a scaling of plasticity only and does not include a scaling of the neural dynamics. The exact values of parameters/gains, throughout the different simulation phases can be found in Table 2.

### Performance metric

Generally speaking, memory performance can be measured in many ways. Popular dual-process theories of episodic memory state that retrieval is contingent on two independent processes, familiarity (providing a sense of recognition) and recollection (recovering events and their context). In recent discussions of MTL function this has often been understood to also imply separate brain areas for each process. However, computational models have shown that both kinds of recognition judgments can, in fact, be simultaneously supported by the same population (Greve et al., 2010).

To limit the scope of this paper, we restrict ourselves to the evaluation of recall performance, which is measured by cueing the system with a studied pattern and measuring the distance between the respective activations after convergence. The distance metric for recall is borrowed from Greve et al. (2010) and described by Equation (11).

After a full simulation run, covering several simulated weeks, recall performance is evaluated for each network separately. These recall rates are then shown to vary against the time between training and testing. As recall of HIP and CTX is contingent on consolidation over time, these plots may also be called consolidation curves. Because patterns are random, and since both replay behavior and resulting memory consolidation are stochastic, 500 simulation runs were averaged to obtain reliable recall rates.

(11) $$\begin{array}{l} d\left(\vec{a},\vec{b}\right)=\frac{1}{2}\left(\text{1}-\frac{\vec{a}\cdot \vec{b}}{\left|\vec{a}\right|\left|\vec{b}\right|}\right)\\ with\vec{a}=s(t=0),\vec{b}=\underset{t\to \infty }{lim}s(t) \end{array}$$

Equation (11) is a recall metric adapted from Greve et al. (2010). We first cue the system with a studied pattern *a*, observe the resulting activation *b* (attractor convergence), and measure the distance *d* between the respective activations in accordance with Equation (11). Studied patterns are expected to have a recall distance near zero, while new patterns will converge to rather distant attractors. We compute an optimal decision boundary for recall judgments (one for each of the three networks) by minimizing the summed type I and type II errors over all possible decision boundaries, similar to Greve et al. (2010). Decision boundary values for each stage can be found in Table 1.

Beyond looking at each of the three networks separately, we could view the model as one integrated memory system and thus disregard the origin of a recalled pattern in quantifying recall. In fact, whether a memory is still dependent on hippocampus, or already fully consolidated into hippocampally independent, neocortical long-term memory, makes no behavioral difference in recall. We thus define an effective combined recall rate, accessing all three networks during the recall phase.

### Simulated lesioning, modulation, and sleep deprivation

To simulate progressing degrees of hippocampal damage, we disable an increasing ratio of HIP units. Disabling a unit also entails nullifying every synaptic connection from or to that unit. To avoid bias in relation to any training pattern, the disabled units were randomly selected. Temporal gradients of amnesia were thereafter measured by comparing the resulting change in recall rates. Anterograde effects were measured by lesioning the system before learning and then comparing the achieved performance of the damaged system against an unlesioned control simulation. Modulations of plasticity were made via a temporal up or down-regulation of learning time constants τ_*L*_, and a scenario of persistent sleep deprivation was implemented by reducing the length of the sleep phase by 50%.

## Results

### Consolidation and amnesia

We ran the entire system in the described simulation cycle (Figure 4) for 39 simulated days and attempted to consolidate a total of 351 memory patterns. The unused patterns of day 40 were used to validate thresholds of the recall metric by measuring false positives (see Equation 11).

**Figure 6:**
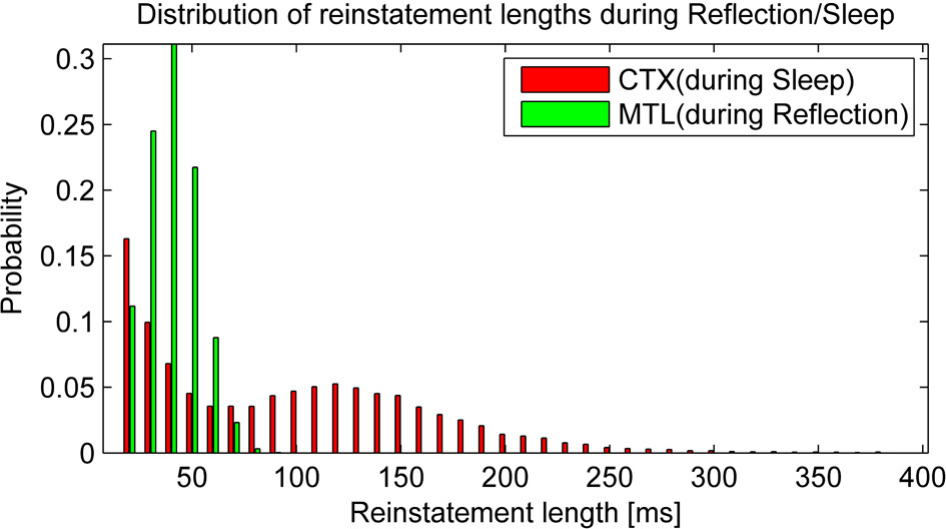
**Replay drives reinstatements of earlier patterns in HIP or CTX, respectively**. The length of these reinstatements is distributed around an average reinstatement length of 40.08 ms for PFC-driven HIP reinstatements during reflection and 95.43 ms for HIP-driven CTX reinstatements during sleep.

Before we take a look at consolidation over time, it is worth taking a glance at the statistics of autonomous reactivation, which is supposed to drive the consolidation process. We classify a pattern as reinstated when the projected activity surpasses 90% overlap with one particular training pattern. After some time, activity of such a pattern will depress below this threshold and eventually new patterns will emerge. We find that reinstatement events occur with a frequency of 6.56 Hz in HIP during reflection and 6.13 Hz in CTX during sleep. It is noteworthy that these events vary in length (Figure 6) due to random correlations between patterns and varying trace strength. While PFC encoding strength is more uniform, yielding a unimodal distribution of HIP reinstatement lengths, this does not hold true for CTX reinstatements during sleep, where we can clearly distinguish between weakly (i.e., briefly) reactivating patterns and strong reactivations with much longer durations, which presumably consolidate better.

**Figure 7:**
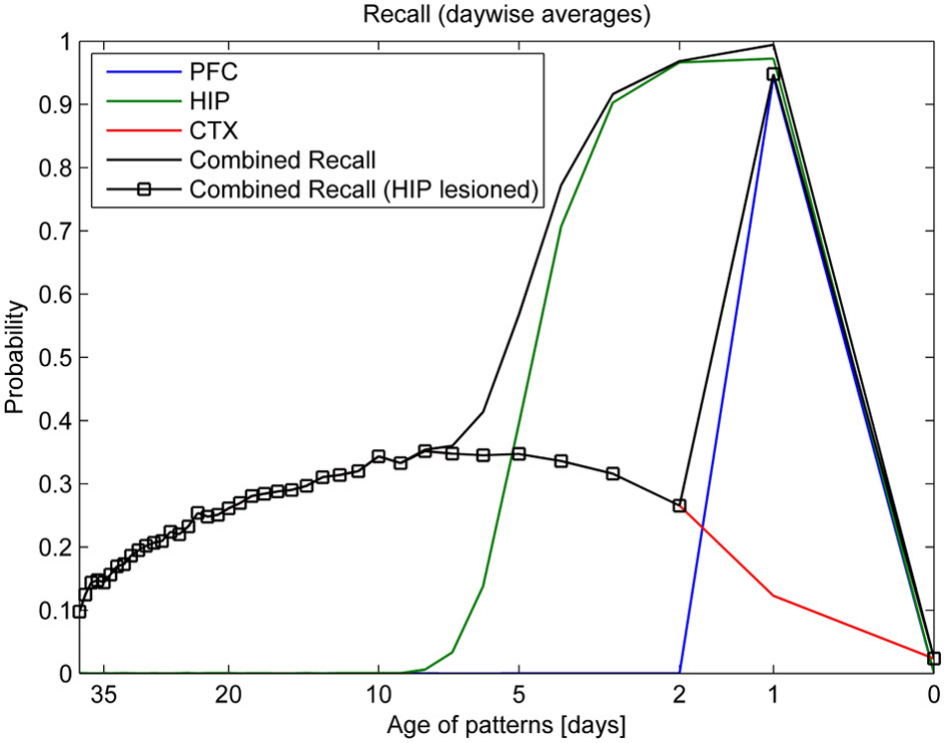
**Consolidation, as measured by recall rates of training patterns from each stage (PFC, HIP, CTX)**. By averaging the recall rates for patterns introduced on the same day, we obtain a more direct relationship between the recall rate and the age of a pattern in days. Combined recall from all stages (solid lines) is shown with and without hippocampus (full lesion) to illustrate its importance for patterns of different age.

Turning our eye to learning, forgetting, and consolidation over time, the top panel of Figure 7 shows that PFC can reliably store only the most recent patterns of the last day (343–351), while HIP can recall much older patterns. Forgetting in CTX is very slow: some of the retrievable patterns are more than a month old. However, only about a third of the patterns shown ever successfully consolidate into retrievable long-term memories. Our analysis shows that consolidation failure is typically rooted in insufficient hippocampal replay during sleep, so familiarity (which could be measured using a different metric, not shown here) is often still established. In conjunction with learning repetition or plasticity modulation, full consolidation (i.e., independent CTX recall) of any specific pattern can, however, be virtually guaranteed (Fiebig, 2012), as we also show in the modulation experiment illustrated in Figure 10. CTX recall of recent patterns is usually weak, as they were not sufficiently consolidated during sleep yet. Maximum cortical consolidation is reached about a week after the initial acquisition.

**Figure 8:**
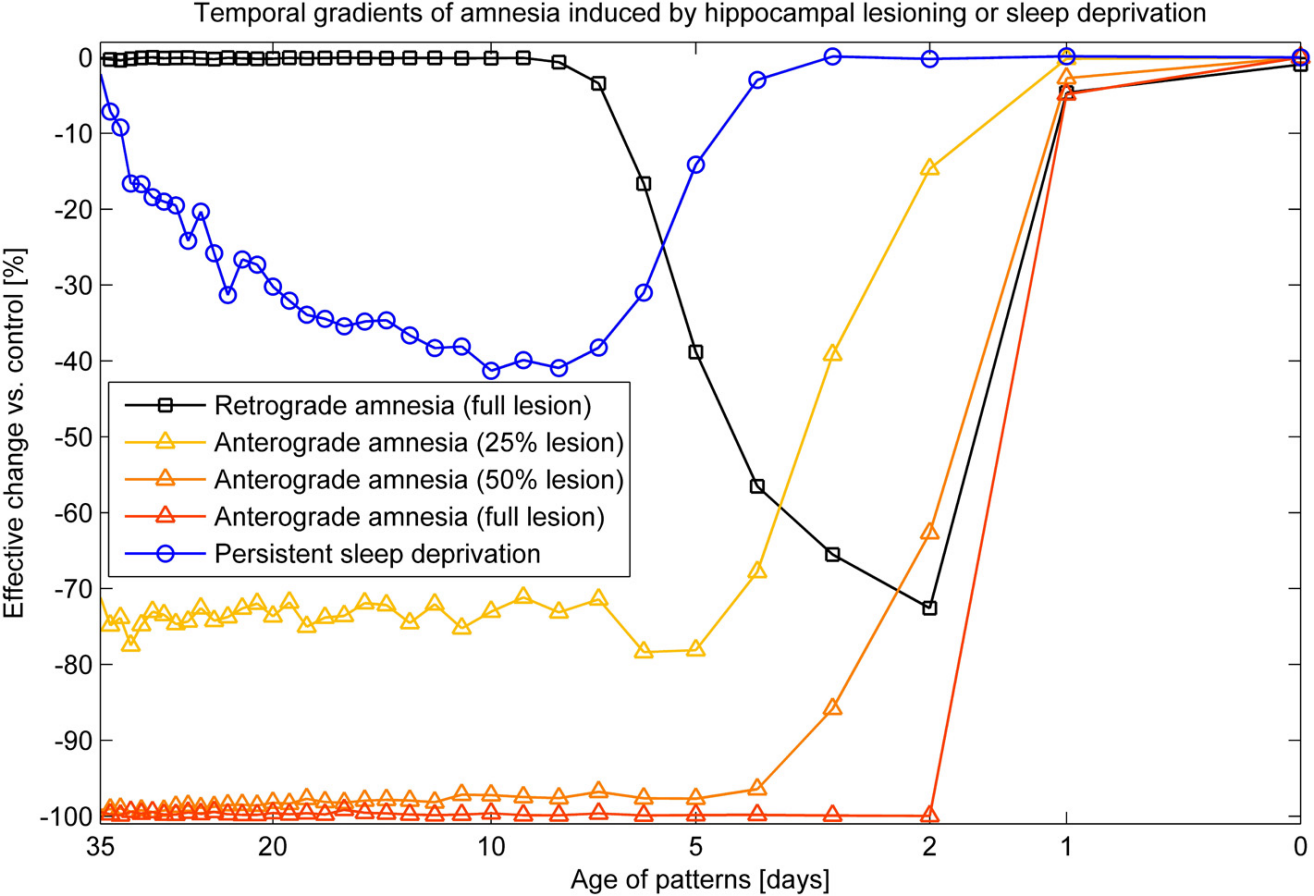
**Five different amnesia gradients**. Retrograde amnesia after full hippocampal lesioning, anterograde amnesia (performance measured after using the lesioned system for 39 days) with different lesion size and persistent sleep deprivation, where we cut the length of the sleeping phase by half.

Combined recall is severely affected when HIP is lesioned, as can be seen in Figure 7. The corresponding RA gradient in Figure 8 shows the ratio of lost recall rate vs. control. On the whole, it shows an inverse temporal gradient. Recall of remove patterns—that have already consolidated—remains unaffected by lesions. Very recent pattern recall is supported by PFC and thus also unaffected by simulated hippocampal lesions. The anterograde gradient shows a persistent, flat deficit (again with the exception of very recent memories) that quickly increases with the size of the lesion, highlighting an increasing inability to form new long-term memories. The onset of amnesia also shifts to more and more recent patterns with greater lesion size, as HIP loses more and more capacity. This kind of amnesia is markedly different from a sleep deprivation experiment shown in the same plot, where reduced sleep-dependent consolidation causes a much less severe anterograde deficit. HIP stays fully functional in this case, so the amnesic effect is seen only much later, when it starts to forget after about a week. This particular finding is inconsistent with biological evidence, which clearly shows impaired hippocampal memory function on many tasks following sleep deprivation, rather than just impaired systems consolidation (Walker and Stickgold, 2006).

**Figure 9:**
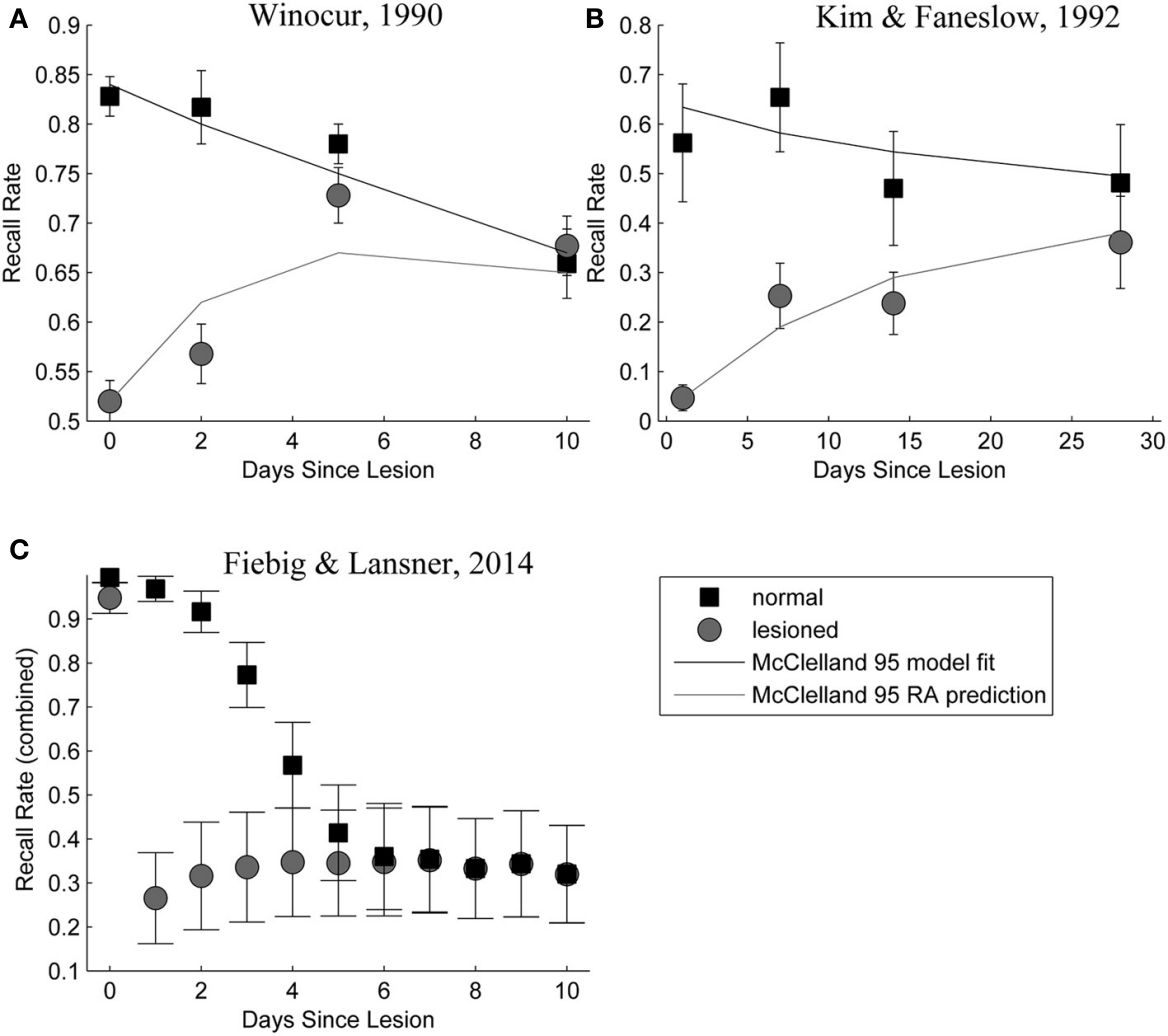
**(A,B)** Behavioral responses of animals receiving extensive hippocampal system lesions (circles) or control lesions (squares) as a function of the numbers of days elapsing between exposure to the relevant experiences and the occurrence of the lesion. Bars surrounding each data point indicate the standard error. Panel **(A)** shows the percentage choice of a specific sample food (out of two alternatives) by rats exposed to a conspecific that had eaten the sample food. Panel **(B)** shows fear (freezing) behavior shown by rats when returned to an environment in which they had experienced paired presentations of tones and footshock. Data in Panel **(A)** are from Winocur (1990). Data in Panel **(B)** are from Kim and Fanselow (1992). The added lines are from a simple differential equations fit from a previous modeling attempt (McClelland et al., 1995). Panel **(C)**: Combined retrieval rates of the normal and hippocampally lesioned simulation model. Rather than the standard error (which is too small to show, as we average 500 simulations), error bars indicate a standard deviation of the underlying data, showing the stochasticity of the consolidation process.

Figure 9 shows a side-by-side comparison of our own simulation results (Figure 9C) and two data sets from rodent experiments (Figures 9A,B), showing temporally graded RA gradients following hippocampal lesioning.

### Modulation experiments

**Figure 10:**
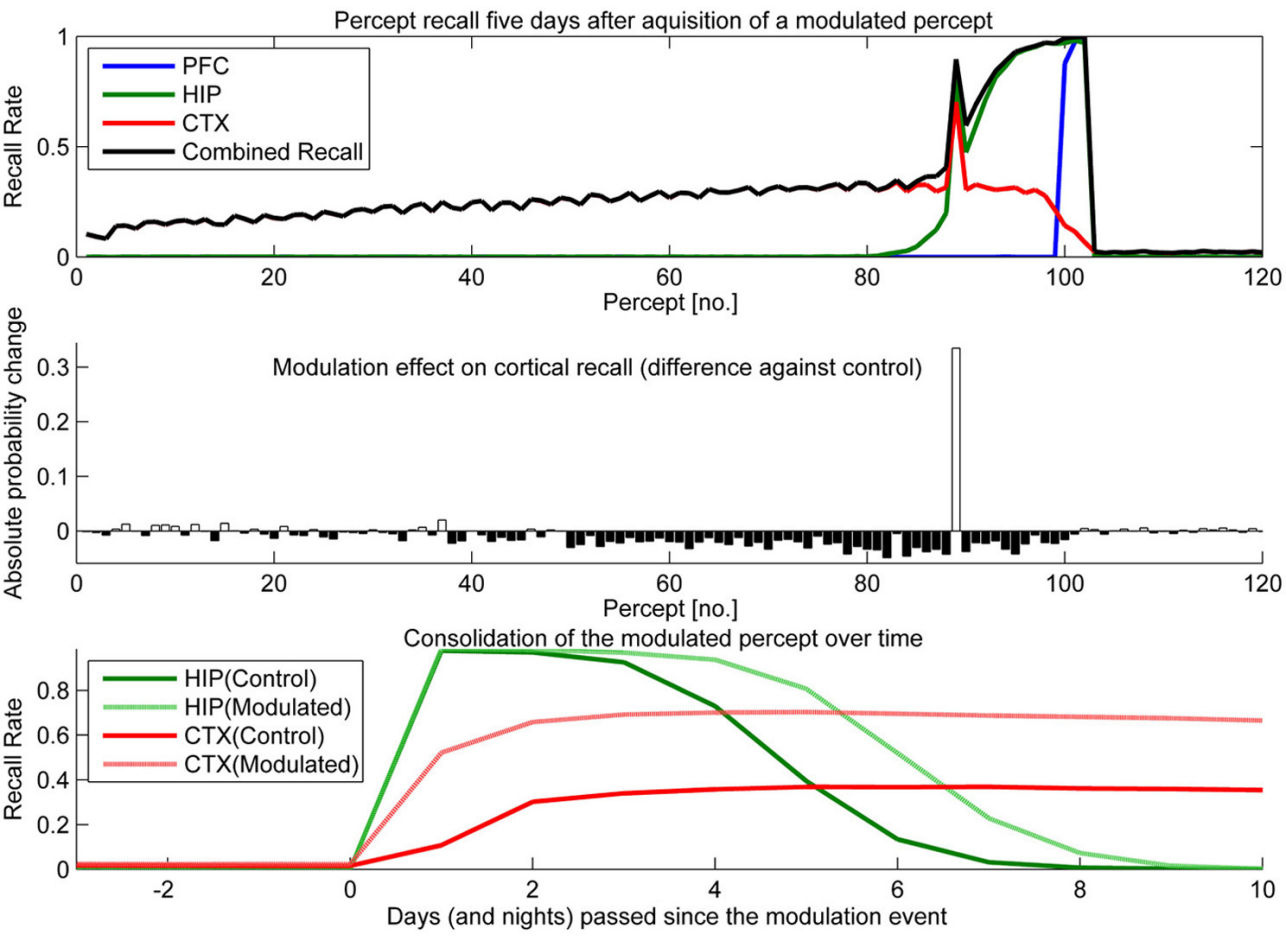
**In this experiment, we boosted hippocampal plasticity during learning of percept 89 (consisting of patterns 265–267) by a factor of two (halfing τ_*L*_) and tested recall 5 days later. Top:** consolidation curves showing the probability of successful recall 5 days after introduction of percept 89. **Middle**: the absolute change of recall probability vs. controls (simulation without any modulation). **Bottom**: the time course of consolidation for the modulated percept, as measured by testing HIP and CTX recall every day following the original learning experience.

**Figure 11:**
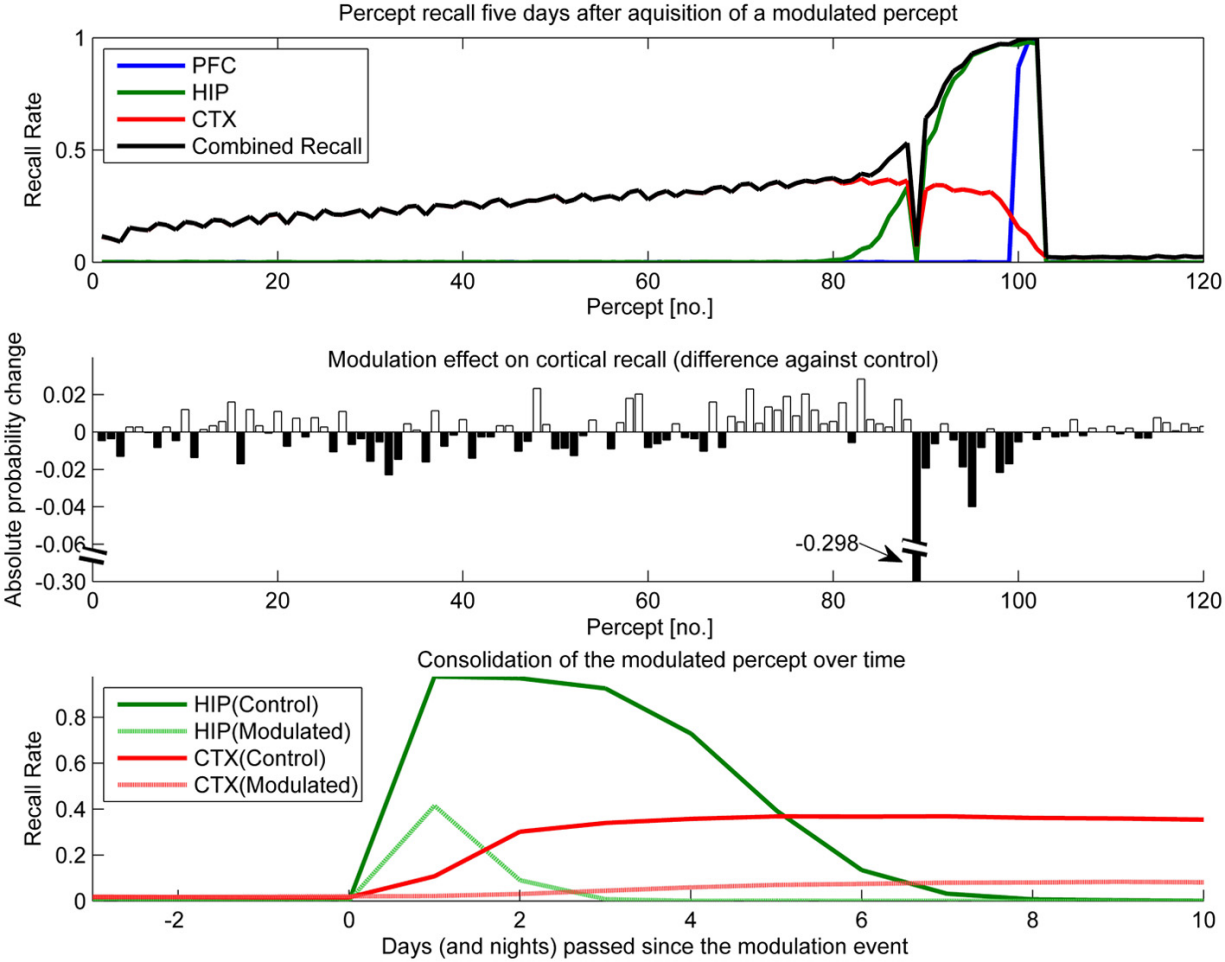
**In this experiment, we simulated the memory impact of triazolam with a half-life of 2 h by reducing hippocampal plasticity by a factor of 10 and decaying this modulation with a 2 h half-life to the original level of plasticity**. The modulation was triggered at the introduction of percept 89. **Top**: consolidation curves measured 5 days after the modulation event, showing the lasting effect on the probability of successful recall. **Middle**: the absolute change of recall probability vs. controls. Note that the y-axis was broken to also visualize the smaller impact seen in the other, unmodulated percepts. **Bottom**: the time course of consolidation for the modulated percept, obtained by testing recall from HIP and CTX every day following the original learning experience.

To test the effect of plasticity modulations on consolidation, we ran two simulations. One had selectively up-regulated plasticity (Figure 10) for one of the percepts shown over the course of a stimulation and the other had a transient down-regulation of plasticity (Figure 11).

Sudden up-regulation of HIP plasticity by a factor of two during learning of one specific percept (modeling something like an emotional relevance signal or attention), can double CTX recall probability in out model, indicating successful consolidation (Figure 10). The middle panel shows that increased recall of this percept comes at the cost of reduced consolidation odds mostly for patterns learned before, but also after. The negative retrograde impact (percept no. <89) is mostly due to HIP overwriting; weakening some patterns to the point that they no longer reactivate/consolidate. However, there is also an additional anterograde effect, which due to the week-long consolidation time window affects a few patterns still in consolidation during the time of modulation (~76–89) as well as many patterns learned after. The lower panel illustrates the extended HIP lifetime of the boosted percept 89, which means that the negative anterograde effect on new patterns stretches out for some time after the modulation occurs (i.e., percepts 90–100 show diminished consolidation vs. control). Note that this occurs because patterns of the modulated percept outcompete other patterns for reactivation during sleep for some time, until HIP strength of percept 89 was diluted to the point that its patterns do not activate preferentially anymore. Very remote percepts are less affected, as their consolidation window has already closed because of hippocampal forgetting. Finally, the bottom panel shows how this modulation not only increases the percepts HIP life-time by 1 or 2 days, but stronger encoding results in both faster and more successful neocortical consolidation. More frequent reactivations during sleep cause maximum neocortical consolidation of the modulated percept after just 3 nights.

The temporal down-regulation of HIP plasticity (Figure 11) yielded a much more peculiar memory effect, namely narrowly focused AA in conjunction with retrograde facilitation. Due to the timing of the modulation onset, the most affected patterns belong to a single percept, which was introduced right at the onset of said modulation. Later precepts were barely affected, due to the fast decay of the modulation. The top panel of Figure 11 shows CTX and HIP recall probability of the most affected percept near zero, indicating that the modulation effectively disrupted hippocampal encoding and subsequently diminished consolidation. The middle panel reveals that in addition to this AA effect, percepts/patterns learned up to 6 days before the modulation exhibit improved consolidation (5 days after the modulation event, all but one of the 20 percepts learned before the event show a positive change in performance vs. control) This retrograde facilitation effect underscores the competitive nature of consolidation during sleep: Because patterns of the blocked percept are encoded so weakly, other, older patterns can reactivate instead during the sleep phases following the modulation, thus improving their consolidation odds. Finally the bottom panel shows how this modulation not only decreases the HIP lifetime of the affected percept to a mere 2 days, but also how weaker encoding results in slower and much reduced neocortical consolidation.

## Discussion

### Addressing the four challenges

We tasked ourselves with four goals: To implement autonomous replay, address the temporal scope of systems consolidation, include working memory in that scope, and defeat the common problem of CF. To these ends, we have built an extended three-stage implementation of the CLS framework using a consolidation chain of Bayesian Confidence Propagation Neural Networks, capable of autonomous replay. Where other models resort to forced activations and top-down control to generate reinstatement dynamics, we have shown that on-going internal activity (autonomous replay) is sufficient for consolidation along a chain of networks with differing memory traces, sparsity, network structure, network size, and most importantly extreme differences in plasticity time-constants, spanning several orders of magnitude. The model thus constitutes an interactive network of diverse recurrent neural networks. (e.g., CTX feeds into HIP, building a sparse hippocampal trace, which in turn facilitates cortical consolidation via back-projections during sleep reactivations).

Our model implements a functional consolidation process from one-shot learning capability to stable neocortical memory engrams due to its three-stage architecture and wide span of time constants. The model parameterizes the mechanism behind different cortical memory systems, from short-term working memory to long-term memory in terms of different set points for plasticity of synaptic weights and intrinsic excitability. Furthermore, the model can keep learning indefinitely and functionally solves the problem of CF by selective, competitive consolidation with simultaneous learning and forgetting on all timescales, a process which also explains why only a fraction of all percepts become long-term memories.

### Biological parallels, differences, and implications

Beyond memory functionality, much of the modeled connectivity can be asserted on biological grounds, yet it can be argued, that this does not hold for the PFC-to-HIP pathway critically used in the reflection phase of the simulation. As we pointed out earlier, there is no known direct pathway of this kind. The strongest influence of the PFC on the hippocampus in primates is indirect through parahippocampal cortices (Otani, 2004), most notably the entorhinal cortex, which feeds into the hippocampus and dentate gyrus (which in turn also feeds into the central hippocampal fields). We have implemented this second pathway in our model in a way that simulates the sparsification and pattern separation observed in experimental data of the dentate gyrus (Leutgeb et al., 2007; Bakker et al., 2008). It is conceivable that the direct PFC-to-HIP connection in our model can be functionally replaced by an indirect pathway through CTX instead. However, the biggest implementation hurdle with respect to this is the achieved sparsification itself, as non-consolidated CTX patterns driven via the PFC will be noisy. This noise becomes most problematically amplified due to pattern separation in the forward connection to HIP, which is otherwise most beneficial in improving HIP capacity and reactivation dynamics during sleep. As this example shows, models of this kind can help us identify architectural problems in neural systems analysis.

Correctly scaled, the model predicts that many hundreds or thousands of reactivations are necessary for guaranteed consolidation. This might seem like a huge number but is, in fact, congruent with biological data: rodent studies have shown average SPW/R event frequencies between 0.3 and 1.2 Hz during SWS, which are significantly increased in number and amplitude after learning and recall (Eschenko et al., 2008). Even a single hour SWS yields more than 10^3^ SPW/R events associated with hippocampal reactivations. A week-long consolidation period thus contains on the order of 10^5^ replay events or more, to be distributed over the select set of consolidating patterns.

Spontaneously occurring HIP reactivations in our model are signified by sharp population activity bursts, occur with a frequency of roughly 6 Hz and last for 30–170 ms, which is similar to biophysically observed sharp-waves that have been closely linked to hippocampal reactivations (see Introduction). The fact that this is achieved with biophysically constrained parameter values, i.e., the adaptation time constant τ_*A*_, adds to the list of interesting biological analogies.

Obviously the real process behind acquisition and consolidation of episodic memory is much more complex than our model suggests, yet despite many simplifications, the results show a range of experimentally observed properties and characteristics. These include competitive consolidation, effects of primacy and recency in short-term consolidation (not specifically discussed here, see Lansner et al., 2013), retrograde facilitation after impaired acquisition, as well as typical amnesia effects following simulated hippocampal lesions.

With respect to the latter, we conclude that the model exhibits temporally graded RA similar to pathologies seen in human case studies, such as Patient HM (Scoville and Milner, 2000): intact working memory, temporally graded RA, preserving remote cortical memories, as well as severe, flat AA. Given that these observations were a major reason for the development of consolidation theory and hippocampal memory research in the first place; our computational model is a rather successful implementation of these concepts. The similarity between our RA curves in Figure 9, bottom panel and those in the top panels, showing experimental RA is striking, confirming predictions about the shape of the amnesia gradient (Nadel and Moscovitch, 1997).

However, our model also exhibits one peculiar difference to the above named experimental studies. It predicts strong recall of very recent patterns, as they are supported by hippocampally-independent working memory. It is necessary to differentiate this prediction of a retrograde gradient from the shown anterograde preservation of working memory capacity following MTL damage encompassing the hippocampus (Jeneson et al., 2010; Jeneson and Squire, 2012). To the authors knowledge, a retrograde preservation of active working memory traces has not been shown before and constitutes a testable prediction, given neurophysiological deactivation of hippocampal function on the timescale of working memory, such as focal cooling may allow (Tanaka et al., 2008). Experimental lesion studies (Squire and Cohen, 1979; Winocur, 1990; Zola-Morgan and Squire, 1990; Kim and Fanselow, 1992) simply cannot account for the fleeting storage of new percepts in short-term memory. For example, test animals (rats, monkeys) are lesioned under deep anesthesia and require several days rest after the lesioning operation. Training, lesioning and directly testing an animal within seconds (the timespan of working memory) is practically impossible. Rather, tests are run on a daily or weekly basis, which thus necessarily excludes short-term memory.

### On competitive consolidation and memory modulation

The nature of consolidation learning in our model is competitive (only one pattern can be reinstated at a time), so it is highly susceptible to memory modulation or learning repetition. Our model predicts that relevant hippocampal memories (meaning more strongly encoded) consolidate faster and more reliably than other memories: when HIP plasticity is modulated by some kind of relevance signal, the resulting change in memory trace strength directly affects the probability of successful long-term consolidation into cortex, as strongly encoded patterns reactivate both longer and more often than other patterns during autonomous replay (Sandberg, 2003; Fiebig, 2012).

Our series of modulation experiments, where we temporarily up- or down-regulated the degree of plasticity in HIP, can be interpreted as simulations of the consolidation impact of dopaminergic relevance signals (say from the amygdala), attention, or the effect of other plasticity modulating agents like benzodiazepines or ethanol. For an example, studies show that both ethanol (Lister et al., 1987; Givens, 1995, 1996) and benzodiazepines like Triazolam (Hinrichs et al., 1984; File et al., 1999; Fillmore et al., 2001) induce a remarkable combination of AA and retrograde facilitation. The hypothesized mechanism for this is impaired acquisition through a suppression of LTP induction in brain areas required for the initial learning, i.e., PFC and HIP (Blitzer et al., 1990). Reduced new learning presumably benefits consolidation of older memories, as the expression of LTP and ongoing consolidation mechanism itself is left intact.

Triazolam has a half-life of about 2 h, and our modeling of a similar, temporally decaying plasticity disruption (Figure 11) yields the same peculiar combination of AA and retrograde facilitation. We consider the successful replication of this effect in a working model based on artificial neural networks a step forward in the modeling of memory consolidation, improving our confidence in working implementations of CLS.

## Conclusion

Contradictory biological evidence regarding disassociations in RA between different aspects of declarative memory (Nadel and Moscovitch, 1997) and evidence of very extensive and sometimes flat RA gradients (Travis et al., 2010) clearly point out weaknesses in the current consolidation model. Similarly, the CLS concept of low cortical involvement during initial acquisition has recently been called into question by experimental studies (Tse et al., 2011). These and other observations underscore. The necessity for testing variations of the model are underscored by these and other observations, including reconsolidation processes (Wittenberg et al., 2002; Alberini, 2005), schema theory (Tse et al., 2007), multiple trace theory (Nadel and Moscovitch, 1997) or a kind of trace-link system (Murre, 1996), some of which have already been shown to deal with certain known inconsistencies of the standard model. Since the conception of CLS, many further details, especially regarding functional disassociations (Eichenbaum et al., 2011) of different parts of the MTL have been explored and deserve further consideration in computational accounts of consolidation.

Irrespective of this, the success of CLS in explaining temporally graded RA, AA, wake and sleep replay and the overall dynamics of memory consolidation even in conjunction with plasticity modulations, underscore its continued scientific value.

A similar network model to the one presented here, but with spiking model neurons is currently under development and with the right tuning and setup, our model can be applied to multiple trace theory or other consolidation mechanisms, such as synaptic reentry reinforcement (Wittenberg et al., 2002) and model REM sleep. For example, we can enable hippocampal reconsolidation by letting HIP stay plastic during replay, such that we not only consolidate neocortical traces during SWS, but replayed hippocampal attractors also reinforce, degrade, or otherwise change themselves with each reinstatement event (Lundqvist et al., 2011). Cascade models of synaptic plasticity (Fusi et al., 2005) as well as further partitioning of the memory system (Roxin and Fusi, 2013) can presumably extend the temporal reach of this model even further.

Our results should be seen as mainly qualitative. McClelland pointed out that the huge range of differences in the timescale of the consolidation phenomenon across species, age and other factors is mostly a function of different learning rates (McClelland et al., 1995). The values of almost all our parameters—including the scaled learning rates—can be questioned on biological grounds. However, our model features a broad array of neurobiological details and clearly shows the viability of a three-stage consolidation chain, driven by autonomous replay that turned attractors into more useful quasi-stable attractors and thus expands the architectural options available to memory researchers looking for appropriate neural network models today.

### Conflict of interest statement

The authors declare that the research was conducted in the absence of any commercial or financial relationships that could be construed as a potential conflict of interest.
